# Contribution of TLR4 signaling in intermittent hypoxia-mediated atherosclerosis progression

**DOI:** 10.1186/s12967-018-1479-6

**Published:** 2018-04-19

**Authors:** Xianqin Zeng, Rong Guo, Mei Dong, Julia Zheng, Huili Lin, Huixia Lu

**Affiliations:** 1 Department of Cardiology, The Second Affiliated Hospital of Fujian Medical University, Quanzhou, 362000 Fujian People’s Republic of China; 2 The Key Laboratory of Cardiovascular Remodeling and Function Research, Chinese Ministry of Education and Chinese Ministry of Health, The State and Shandong Province Joint Key Laboratory of Translational Cardiovascular Medicine, Department of Cardiology, Qilu Hospital of Shandong University, No. 107, Wen Hua Xi Road, Jinan, 250012 Shandong China; 3 Rutgers Robert Wood Johnson Medical School, New Jersey, New Brunswick USA; 4 Department of Cardiology, Ji’an Municipal Center People’s Hospital, Ji’an, Jiangxi China

**Keywords:** Atherosclerosis, Intermittent hypoxia, Toll-like receptor 4, Nuclear factor κappa B, Inflammation

## Abstract

**Background:**

Intermittent hypoxia (IH), a typical character of obstructive sleep apnea (OSA), is related to atherogenesis. However, the role of IH on atherosclerosis (AS) progression and the mechanisms involved remains poorly understood.

**Methods:**

In the present study, high-fat fed ApoE^−/−^ mice were treated with recombinant shRNA-TLR4 lentivirus and exposed to IH. Atherosclerotic lesions on the en face aorta and cross-sections of aortic root were examined by Oil-Red O staining. The content of lipids and collagen of aortic root plaques were detected by Oil-Red O staining and Sirius red staining, respectively. The TLR4, NF-κB p65, α-SMA and MOMA-2 expression in aorta and IL-6 and TNF-α expression in the mice serum were also detected.

**Results:**

Compared with the Sham group, the IH treated group further increased atherosclerotic plaque loads and plaque vulnerability in the aortic sinus. Along with increased TLR4 expression, enhanced NF-κB activation, inflammatory activity and aggravated dyslipidemia were observed in the IH treated group. TLR4 interference partly inhibited IH-mediated AS progression with decreased inflammation and improved cholesterol levels. Similarly, in endothelial cells, hypoxia/reoxygenation exposure has been shown to promote TLR4 expression and activation of proinflammatory TLR4/NF-κB signaling, while TLR4 interference inhibited these effects.

**Conclusions:**

We found that the IH accelerated growth and vulnerability of atherosclerotic plaque, which probably acted by triggering the activation of proinflammatory TLR4/NF-κB signaling. These findings may suggest that IH is a risk factor for vulnerable plaque and provide a new insight into the treatment of OSA-induced AS progression.

## Background

Atherosclerosis (AS) is a chronic vascular disease. High vulnerability of AS plaque is a common pathologic change of acute coronary syndrome (ACS) and stroke, which account for a large part of deaths [1]. Cardiovascular risk factors such as diabetes mellitus, dyslipidemia and hypertension, contribute to AS development. In recent decades, obstructive sleep apnea (OSA) has been recognized as a new risk factor of AS [2, 3]. Patients who suffered from both AS and OSA suffer even worse outcomes [4].

OSA, induced by intermittent partial or complete obstruction of the upper airway during sleep, is a public health problem as it affects one in five adults in western countries [5]. Epidemiological studies have shown a positive association between OSA and AS [6–8]. OSA was also reported to play an important role in accelerating AS plaque formation. The most prominent pathophysiological feature of OSA is intermittent hypoxia (IH), which results from repeated episodes of upper airway obstruction. Accumulating pieces of evidence show a correlation between IH and AS [9–12]. A study has found that IH can trigger multi-pathogenic factors, especially inflammation and dyslipidemia [10]. However, whether IH is related to plaque development and vulnerability is still not fully clear, and the detailed mechanisms involved remain unknown.

Chronic inflammation is now regarded as a common pathway for deteriorating AS plaque [13]. Toll-like receptor 4 (TLR4), a typical representative of pattern-recognition receptors in innate immune responses [14], plays an important role in activation of inflammation in AS. TLR4 signaling in AS is implicated in activation of inflammation and lipid accumulation [15], which are all associated with plaque progression and vulnerability. Furthermore, several studies indicated that TLR4 signaling may be involved in the IH-triggered inflammation [16, 17]. Patients with OSA were also found to have higher expression of TLR4 on monocytes [18]. Nevertheless, it is still unclear whether activation of the TLR4 signaling participates in IH-induced AS progression and vulnerability of plaque.

In this study, we investigated the role of TLR4 signaling in IH-mediated AS progression in an apolipoprotein E-deficient (ApoE^−/−^) mouse model.

## Methods

### Experimental animals

Sixty ApoE^−/−^ mice on the C57BL/6 background (Male, 8 weeks old) were purchased from Beijing Vital River Laboratory Animal Technology Co., Ltd. (Beijing, China). Mice were fed on a high-fat diet (HFD) (0.25% cholesterol and 15% cocoa butter) starting at 8 weeks of age.

### Recombinant lentivirus and gene transfection

Three short hairpin RNA (shRNA) sequences targeting mouse TLR4 gene (NM_021297.3) were designed and constructed. After RNAi screening, the most effective shRNA sequences (sense: 5′-GATCC GCACT CTTGA TTGCA GTTTC ATTCA AGAGA TGAAA CTGCA ATCAA GAGTG CTTTT TTG-3′ anti-sense: 5′-AATTC AAAAA AGCAC TCTTG ATTGC AGTTT CATCT CTTGA ATGAA ACTGC AATCA AGAGT GCG-3′), which exhibited 89.5% of TLR4mRNA reduction, was selected for the study. Recombinant shRNA-TLR4 lentivirus (LV-TLR4i) was generated and purified. LV-enhanced green fluorescent protein (LV-EGFP) viral suspension was prepared, which was purchased from Geneche (Shanghai, China).

**Fig. 1:**
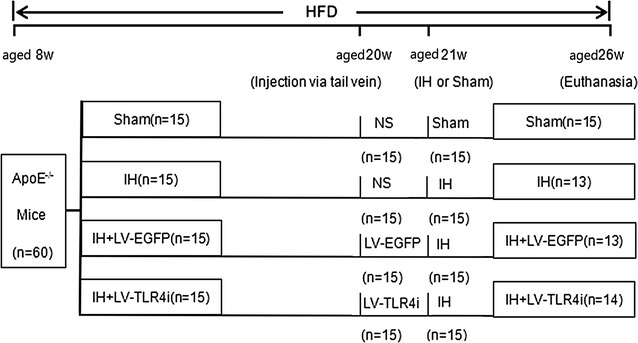
Timeline of the experimental protocol in vivo. *HFD* high-fat diet, *w* weeks, *Sham* continuous air, *IH* intermittent hypoxia, *LV* lentivirus, *LV-EGFP* LV-enhanced green fluorescent protein, *LV-TLR4i* TLR4 gene interference (TLR4i) mediated by lentivirus, *NS* normal saline

All ApoE^−/−^mice were randomly divided into four groups: Sham (n = 15), IH (n = 15), IH + LV-EGFP (lentivirus-enhanced green fluorescent protein) (n = 15) and IH + LV-TLR4i (TLR4 interference mediated by lentivirus) (n = 15) groups. The animal grouping and timeline of the experimental protocol are presented in Fig. 1. At 20 weeks of age, IH + LV-EGFP and IH + LV-TLR4i group mice were injected respectively with LV-EGFP and LV-TLR4i (2.0 × 10^7^ infection unit per mouse) through the tail vein, while normal saline was injected into the other two groups of mice which served as vehicle controls.

### IH exposure

One week after gene transfection, mice were exposed to either Sham or IH. A computer-controlled solenoid valve system was used to control the oxygen concentration by regulating the infusion of nitrogen, room air and oxygen into the exposure chamber. During each cycle of IH, the fractional oxygen concentration of the exposure chamber was reduced to a nadir of 5.5–6.5%, stabilized at that level for 10 s and increased to 20–21% in the subsequent 30 s [9, 10, 16]. The Sham group mice were exposed to a similar environment but only room air was used during the IH exposure time (08:00–17:00 daily) to coincide with the animals’ diurnal sleep period.

Oxygen concentration was measured by oxygen Detector AR8100 (SMART SENSOR, Hong Kong).

### Blood pressure

At these two time points of Pre- and Post-IH, blood pressure was measured by the tail-cuff method (CODA2, Kent Scientific, Torrington, CT) [19].

### Tissue preparation and collection

At the end of exposure protocols, mice fasted for 8 h before euthanasia. Arterial blood was obtained by direct cardiac puncture under 1% pentobarbital anesthesia. The heart with the aortic root, liver, kidney and the artery from the aortic arch to the left and right common iliac artery were quickly harvested and fixed in 4% paraformaldehyde (pH 7.0–7.4) or frozen in liquid nitrogen. Serial cryostat Sects. (5 μm thickness) of the aortic root were prepared by the freezing microtome (Leica CM1950, Nussloch, Germany), and were used for histological and immunohistochemical staining.

### Atherosclerotic lesions and immunohistochemistry analysis

To measure overall load and distribution of AS in the inner wall of the aorta, en face lesion was stained with Oil-Red O. Moreover, the cryostat sections were stained with hematoxylin and eosin (H&E) according to a standard protocol of our laboratory. Oil-Red O staining and Sirius red staining were used to detect the content of lipids and collagen of aortic root plaques, respectively. TLR4, NF-κB p65 (nuclear factor κappa B p65), α-SMA (α-smooth muscle actin), MOMA-2 (macrophages/monocytes) expression in aortic root were detected by immunohistochemical staining. Briefly, cryosections were prepared, hydrated, blocked in blocking solution, and incubated with anti-TLR4 (Abcam, ab120684-1, 1:200), anti-p65 (Abcam, ab7970, 1:250), anti-α-SMA (Abcam, ab5694; 1:200), anti-MOMA-2 (Abcam, ab33451, 1:250) as we previously described [20]. Histological and immunohistochemical staining were analyzed with Image Pro-Plus 6.0. (IPP 6.0, Media Cybernetics, Rockville, MD, USA). Atherosclerotic plaque instability index was calculated according to the standard plaque stabilization score formula: (Oil Red O^+^ area plus MOMA-2^+^ area)/(α-SMA^+^ area plus collagen I^+^ area) [21].

### Biochemical assays

Serum lipid profiles, including serum total cholesterol, low-density lipoprotein cholesterol (LDL-C), high-density lipoprotein cholesterol (HDL-C), total triglycerides (TG) and glucose concentrations were measured by enzymatic assay using an automatic biochemical analyzer (Roche Cobas Integra 800, Basel, Switzerland).

### Enzyme-linked immunosorbent assay (ELISA)

Mice serum levels of interleukin-6 (IL-6) and tumor necrosis factor alpha (TNF-α) were measured using ELISA kits (eBiosciences) according to the instructions.

### Cell culture and treatment protocol

Human umbilical vein endothelial cells (HUVECs) were purchased from CHI SCIENTIFIC Biotechnology (Jiangyin, China). Cells were cultured in Dulbecco’s modified Eagle medium (DMEM) supplemented with 10% fetal bovine serum (FBS; Gibco Inc.), penicillin (100 U/mL) and streptomycin (100 μg/mL).

Before exposure to hypoxia/reoxygenation (H/R), we first performed cell transfection. Lentivirus expressing either nonsense shRNA or anti-TLR4 shRNA were transfected into the HUVECs. HUVECs were plated at a density of 3 × 10^5^ cells per 35 mm plate and cultured for 12 h before transfection. Then medium containing packaged lentivirus was added and 12 h later was replaced with fresh medium. After 48 h, the interference effect of TLR4 protein was detected by using western blot for each experiment.

Hypoxia was induced in a Whitley H35 Hypoxystation equipment of CO_2_/O_2_ incubator for hypoxia research. Briefly, cells were cultured in 35 mm dishes at 1% O_2_ for 6 h and then reoxygenationed at the time periods of 2, 12 and 24 h, respectively. Control cells were cultured in normoxia in the same incubator and harvested at the specified times.

### Western blot analysis

Proteins were extracted from treated HUVECs and ApoE^−/−^ mice aortas. All samples were lysed on ice for 30 min in a RIPA buffer (Beyotime, Nantong, China) after being ground with a pestle. Equal amounts of protein were separated by SDS-PAGE and transferred to PVDF membranes. After blocking by 5% non-fat milk, the membranes were incubated with primary antibodies for TLR4 (Abcam, ab120684-1, 1:500), p65 (Abcam, ab7970, 1:1000), interleukin-1β (IL-1β)(Abcam, ab9722, 1:500) and p-p65 (Ser536) (Cell signaling, #3033, 1:1000) overnight at 4 °C. After washing and incubating with an HRP-conjugated secondary antibody (1:10,000), the immunoreactive bands were visualized using chemiluminescence (Millipore Corporation, Billerica, MA, USA). Sample loadings were normalized to β-actin expression.

### Quantitative real-time PCR (qRT-PCR)

Total RNA was extracted using RNAiso Plus reagent (TaKaRa Biotech Corporation, Dalian, China) and treated with RNase-free gDNA Eraser to remove traces of genomic DNA. Complementary DNA was generated with a PrimeScript™ RT reagent kit with gDNA Eraser (TaKaRa Biotech Co.). A SYBR^®^ Premix Ex Taq™ Kit (TaKaRa Biotech Co.) was used for a real-time polymerase chain reaction (PCR) reaction examination. The sequences of primers (5′–3′) were (1) for TLR4: ATGGC ATGGC TTACA CCACC (forward) and GAGGC CAATT TTGTC TCCAC A (reverse); (2) for β-actin: CACTG TGCCC ATCTA CGA (forward) and GTAGT CTGTC AGGTC CCG (reverse). The relative expression of the target gene was normalized against β-actin and the data were analyzed by the 2^−ΔΔCT^ method [22].

### Statistical analysis

The data are shown as mean ± SEM. Continuous data for two group differences were analyzed by Student’s t test and differences among groups were analyzed by one-way ANOVA with post hoc analysis. A p value < 0.05 was considered statistically significant. Data were analyzed using SPSS 17.0 software (SPSS Inc., Chicago, IL, USA).

## Results

### IH exposure accelerated atherosclerotic plaque growth relying on the expression of TLR4 in ApoE^−/−^ mice

**Fig. 2:**
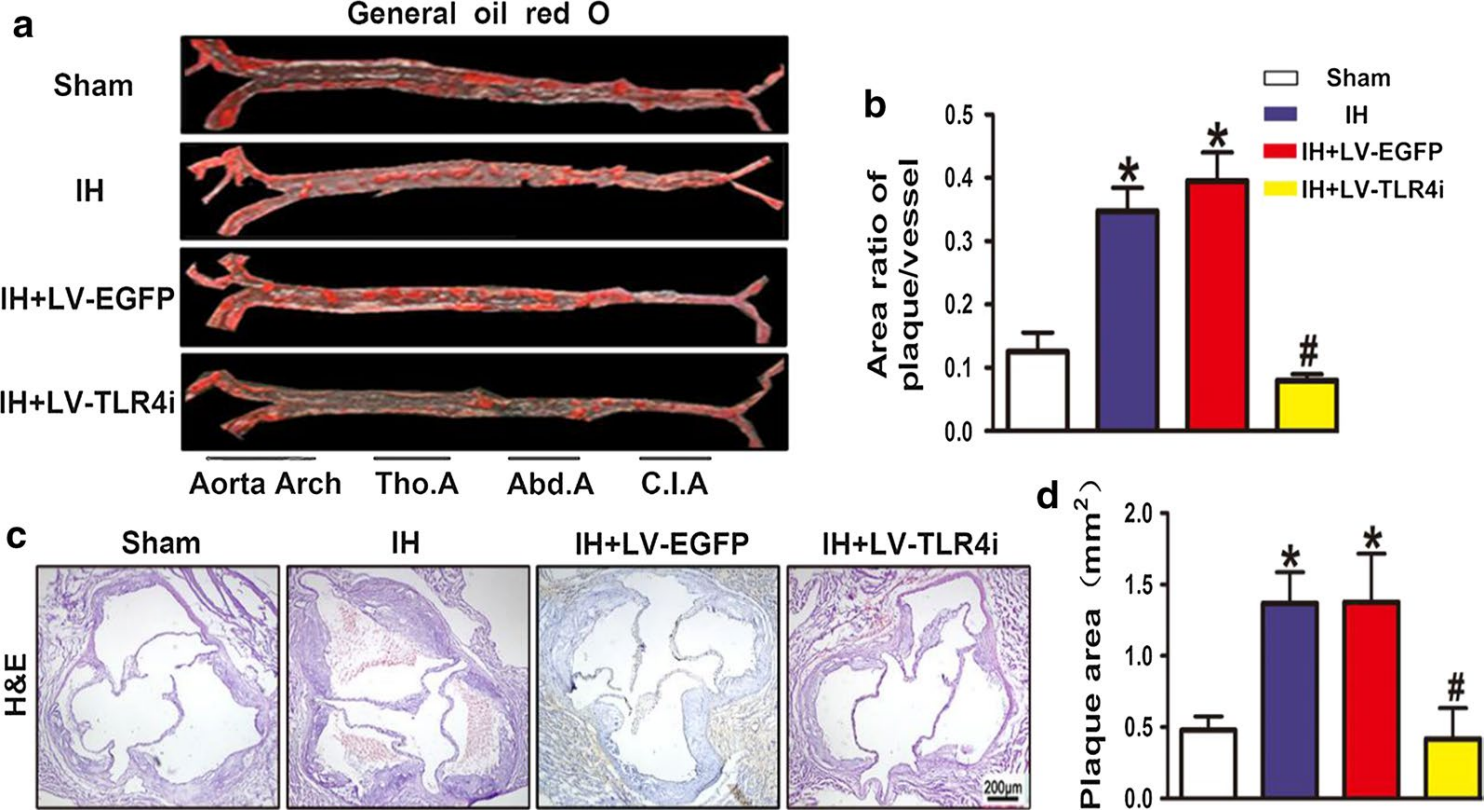
Atherosclerotic plaque growth in ApoE^−/−^ mice. **a**, **b** General oil red O-stained aortas and the area ratio of plaques of four groups. **c**, **d** H&E stained sections of aortic root and the quantification of cross-sectional areas of plaque area in aortic roots. Data represents mean ± SEM of four separate samples.*p < 0.05 versus Sham group and ^#^p < 0.05 versus IH + LV-EGFP group. *Tho.A* thoracic artery, *Abd.A* abdominal artery, *C.I.A* common iliac artery

A growing number of in vivo studies show that IH exposure triggers multiple proatherogenic factors [9–12]. However, the impact of IH on plaque growth and its underlying mechanisms remains poorly understood. Thus, we observed the consequence of IH exposure in affecting atherosclerotic plaque growth by using special staining techniques. As expected, general Oil Red O staining showed that arteries, crossing from the aorta arch to the common iliac artery, contained extensive atherosclerotic lesions throughout the artery trunk after 5-weeks of IH exposure (Fig. 2a, b). Moreover, the average size of plaque areas in the IH group was increased 2.5- to threefold than in the Sham group (Fig. 2c, d).

To further explore whether TLR4 signaling was involved in IH-mediated atherosclerotic plaque growth, mice were transfected with TLR4 specific shRNA and then treated with IH exposure. Compared with the IH + LV-EGFP group, the IH + LV-TLR4i group mice showed fewer atherosclerotic lesions (Fig. 2a, b) and smaller plaque areas (Fig. 2c, d). Therefore, these findings showed that TLR4 signaling was involved in IH-triggered atherosclerotic plaque growth.

### IH-mediated TLR4 expression promoted atherosclerotic plaque instability in vivo

**Fig. 3:**
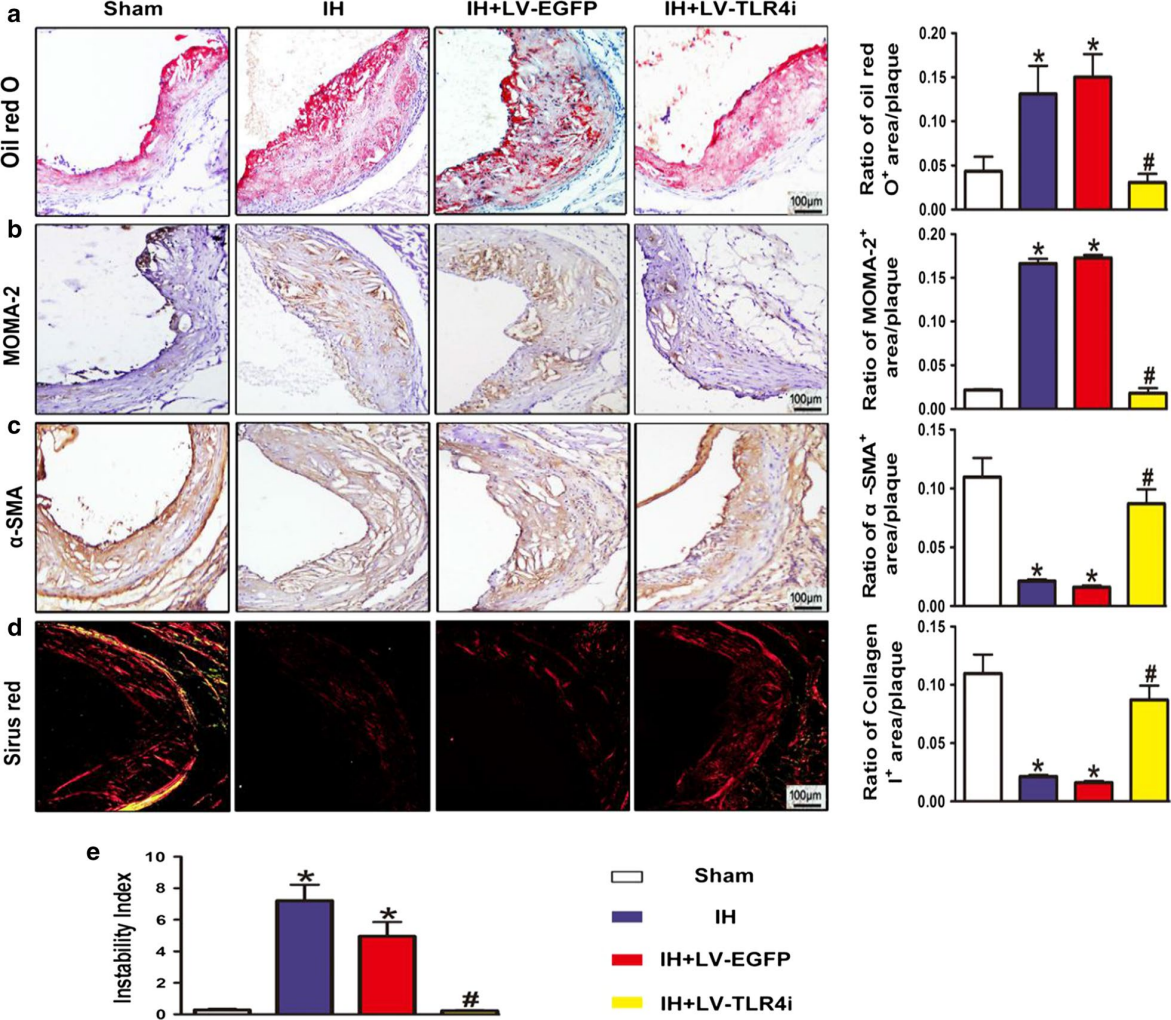
Instability of atherosclerotic plaque. **a** OilRed O staining image and quantification of lipid deposition. **b** Quantification of plaque macrophage content by immunohistochemical MOMA-2 staining. **c** Quantification of plaque smooth muscle cell content by immunohistochemical α-SMA staining. **d** Picrosirius Red staining image and quantification of plaque collagen I content. **e** Plaque vulnerability index [(Oil Red O + area plus MOMA-2 + area)/(α-SMA + area plus collagen I^+^ area)] in atherosclerotic plaque. Data represent mean ± SEM of four separate samples.*p < 0.05 versus Sham group and ^#^p < 0.05 versus IH + LV-EGFP group

Finding that IH accelerated atherosclerotic plaque growth in ApoE^−/−^ mice, we further investigated the effect of IH on atherosclerotic plaque vulnerability. A vulnerable plaque is characterized by a large lipid core covered with a thin fibrous cap, containing sparse smooth muscle cells and extensive macrophages [23]. In the present study, IH exposure increased the content of macrophages and lipids but reduced that of smooth muscle cells and collagen I in the atherosclerotic plaque, thus contributing to increased plaque vulnerability in ApoE^−/−^ mice (Fig. 3a–d). The plaque vulnerability index in the IH group was increased in several times relative to the Sham group (Fig. 3e). However, mice transfected with TLR4-specific shRNA showed a more stable atherosclerotic plaque (Fig. 3a–e). These changes in the composition of plaques indicated that IH exposure promotes plaque instability and the potential for plaque rupture. In addition, TLR4 interference modified the composition of plaques and improved the stability of vulnerable plaque in IH-exposed ApoE^−/−^ mice.

### IH exposure exacerbated inflammatory response via TLR4-mediated signaling in ApoE^−/−^ mice

**Fig. 4:**
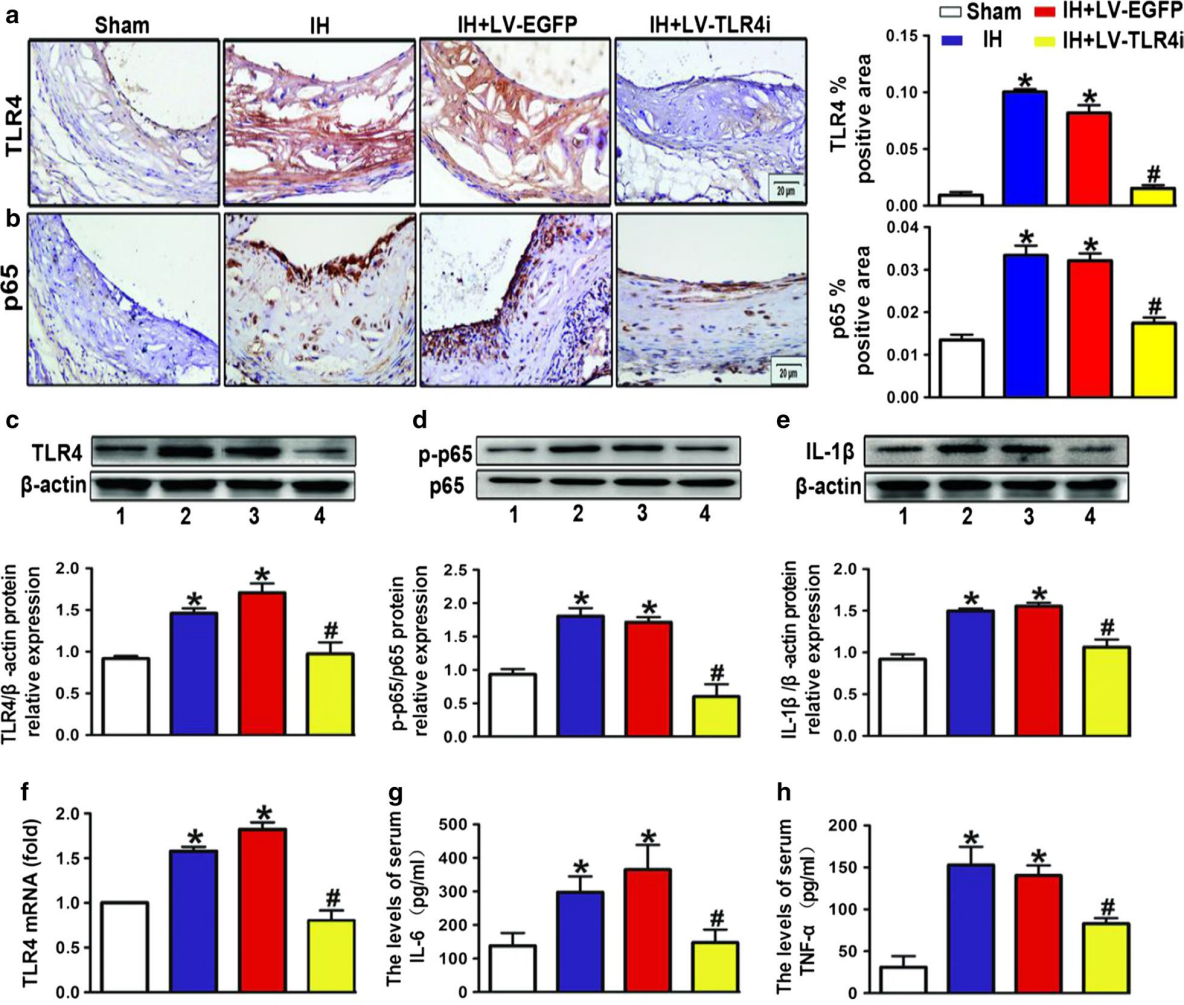
Inflammatory response via TLR4-mediated signaling in ApoE^−/−^ mice. **a**, **b** Quantification of immunohistochemical staining for TLR4 and p65 in atherosclerotic lesions. Negative controls replaced primary antibody with non-immune IgG (Abcam). **c**–**e** Western blot images and quantification of TLR4, p65 and IL-1β expression. **f** RT-PCR analysis of mRNA expression of TLR4 in the vessels. **g**, **h** IL-6 and TNF-α levels determined by ELISA. All quantitative data are mean ± SEM of three separate samples. Bar 1: Sham; bar 2: IH; bar 3: IH + LV-EGFP; bar 4: IH + LV-TLR4i; *p < 0.05 versus Sham group and ^#^p < 0.05 versus IH + LV-EGFP group

TLR4 signaling is closely involved in the activation of the inflammatory response. To investigate whether TLR4 signaling involved in IH-mediated plaque development by aggravating inflammatory activity in vivo, we first examined TLR4 expression in the aortic plaque in IH-exposed ApoE^−/−^ mice. IH upregulated both protein (Fig. 4a, c) and mRNA (Fig. 4f) levels of TLR4 in the aorta. Meanwhile, the nuclear localization of p65 (Fig. 4b) and the phosphorylation of p65 (Fig. 4d) in the IH group were significantly increased than that in the Sham group. Moreover, IH upregulated the protein level of IL-1β (Fig. 4e) and the infiltration of macrophages (Fig. 3b) in atherosclerotic plaque, as well as increased serum levels of IL-6 and TNF-α in ApoE^−/−^ mice (Fig. 4g, h).

To explore possible mechanisms by which IH promoted the inflammatory response, we further elaborated the relationship between TLR4 signaling and inflammatory activity in IH-exposed ApoE^−/−^ mice. Compared with the IH + LV-EGFP group, TLR4 interference evidently reduced TLR4 expression (Fig. 4a, c, f) and activation of p65 (Fig. 4b, d) in the IH + LV-TLR4i group mice aortas. Moreover, inhibition of TLR4 could restore IL-1β, IL-6, and TNF-α to the levels of the Sham group (Fig. 4e, g, h). Likewise, TLR4 interference effectively blocked the increasing of macrophage infiltration in IH-exposed atherosclerotic plaque (Fig. 3b). Consequently, we concluded that IH accelerated plaque progression via TLR4-triggered vascular and systemic inflammatory responses.

### TLR4 interference restored IH-induced body weight loss but had no effect on blood pressure elevation in ApoE^−/−^ mice

The results of body weight and blood pressure of the four groups are presented in Table 1. There are no differences in body weight among the four groups at baseline (V_0_). After 5 weeks of IH exposure, IH and IH + LV-EGFP groups were significantly reduced in body weight (V_1_) compared with the Sham group (Table), but this effect of IH on body weight was reversed by TLR4 interference, as shown in Table 1.

**Table 1:** Body weight, weight loss and systemic blood pressure in ApoE^−/−^ mice

Groups	Body weight (g)			Blood pressure (mmHg)			
	Pre-IH	After-IH	WL	Pre-IH	Pre-IH	After-IH	After-IH
–	(V0)	(V1)	(V0 − V1)	(SBP)	(DBP)	(SBP)	(DBP)
Sham	30.77 ± 0.47	28.94 ± 1.06	1.84 ± 0.81	116.67 ± 2.56	73.90 ± 3.81	102.89 ± 0.90	59.67 ± 1.96
IH	30.64 ± 0.31	24.57 ± 0.74*	6.06 ± 0.69*	116.47 ± 1.79	73.24 ± 1.19	129.89 ± 2.11*	81.07 ± 4.05*
IH + LV-EGFP	30.79 ± 0.28	24.39 ± 0.93*	6.40 ± 1.14*	121.90 ± 2.52	71.62 ± 2.27	123.11 ± 2.42*	76.77 ± 2.99*
IH + LV-TLR4i	31.57 ± 0.42	30.36 ± 0.55^#^	1.21 ± 0.38^#^	123.43 ± 2.89	77.47 ± 2.35	127.00 ± 2.87*	80.00 ± 2.94*

Results are expressed as mean ± SEM and n = 6 per group

*WL* weight loss, *V0* pre-IH body weight, *V1* after-IH body weight

* p < 0.05 versus Sham group and ^#^ p < 0.05 versus IH + LV-EGFP group

At baseline, there were no differences in systolic blood pressure (SBP) and diastolic blood pressure (DBP) among the four groups. After IH exposure, SBP and DBP were significantly increased compared with the Sham group. However, there were no significant differences between the IH + LV-TLR4i group and the IH + LV-EGFP group (Table 1), indicating that TLR4 interference had little effect on IH-elevated blood pressure.

### The role of TLR4 in IH-induced glycolipid disorders in vivo

**Fig. 5:**
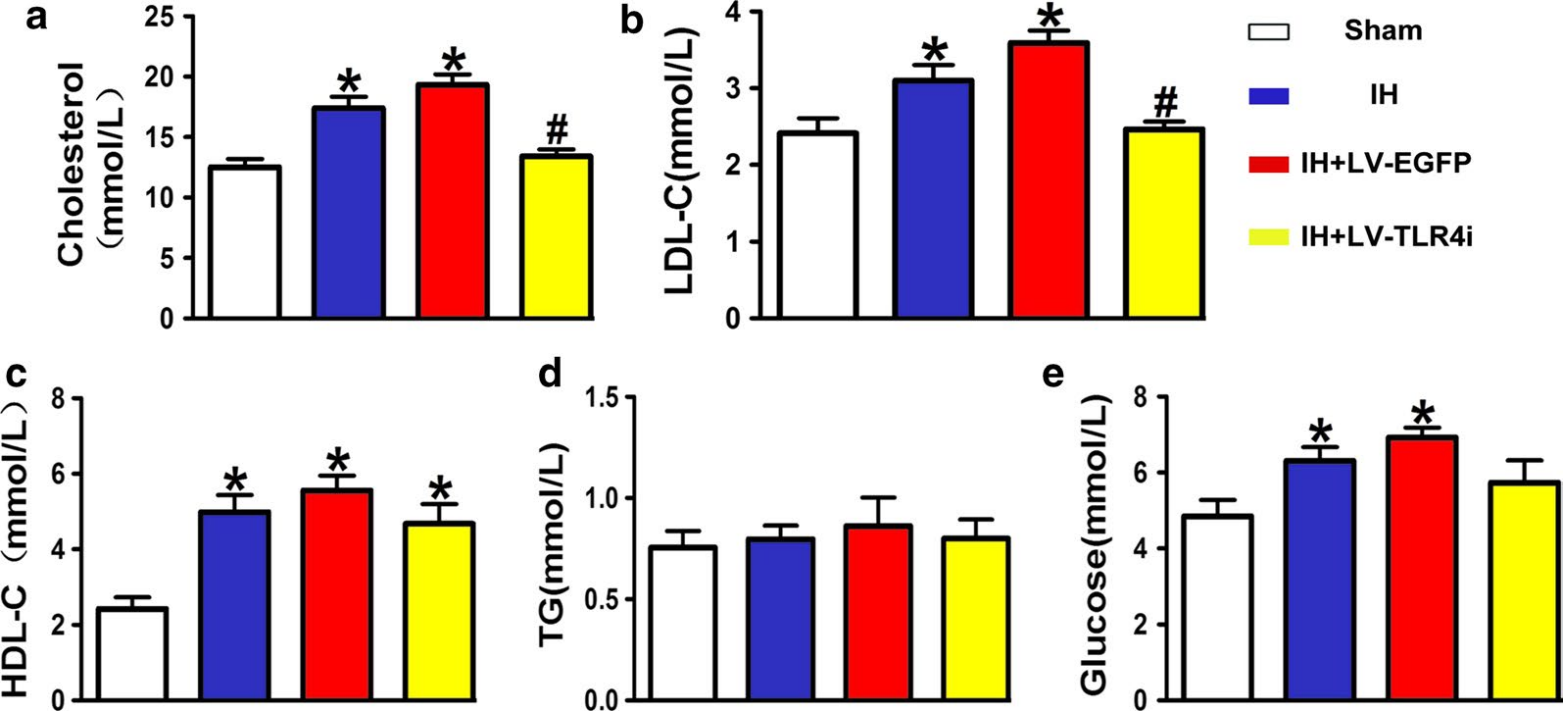
Glycolipid disorder in vivo. **a**–**e** Total cholesterol (n = 6), high-density lipoprotein cholesterol (HDL-C) (n = 6), low-density lipoprotein cholesterol (LDL-C) (n = 6), triglycerides (TG) (n = 6) and blood glucose (n = 6) in ApoE^−/−^ mice. *p < 0.05 versus Sham group and ^#^p < 0.05 versus IH + LV-EGFP group

As most of the OSA patients are complicated with dyslipidemia, we investigated whether IH had an effect on glycolipid metabolisms. As shown in Fig. 5a–e, IH significantly increased serum levels of total cholesterol, LDL-C, HDL-C, glucose, with no influence on TG levels. TLR4 interference reversed the IH-induced increase of total cholesterol and LDL-C, but had little influence on the serum levels of HDL-C, TG and glucose. Therefore, IH could cause glycolipid metabolism disorders and TLR4 was involved in IH-mediated hypercholesterolemia.

### Essential role of TLR4 in H/R activated proinflammatory signaling in vitro

**Fig. 6:**
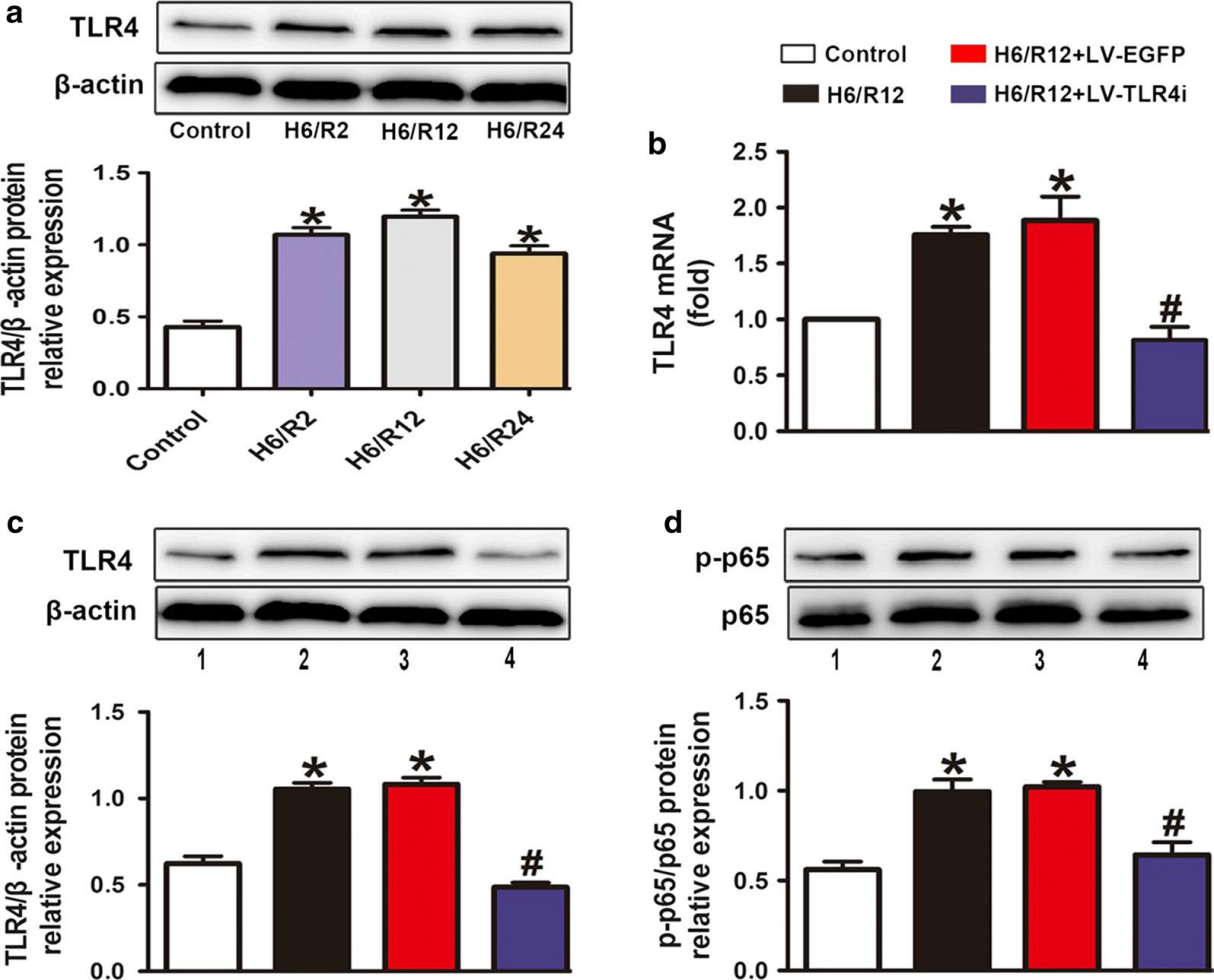
Essential role of TLR4 in H/R exposure activated proinflammatory signaling in vitro. **a** Western blot images and quantification of TLR4 in HUVECs with distinct time periods of reoxygenation exposure. **b** Effects of H/R exposure induced mRNA expression of TLR4 and TLR4 interference reversed these effects in HUVECs. **c**, **d** Western blot images and quantification of TLR4 and p-p65 expression in treated HUVECs. All quantitative data are mean ± SEM of three separate samples. Bar 1: Control; bar 2: H6/R12; bar 3: H6/R12 + LV-EGFP; bar 4: H6/R12 + LV-TLR4i; *p < 0.05 versus Control group and ^#^p < 0.05 versus H6/R12 + LV-EGFP group. H6/R2, Reoxygenation 2 h after 6 h of hypoxia; H6/R12, Reoxygenation 12 h after 6 h of hypoxia; H6/R24, Reoxygenation 24 h after 6 h of hypoxia

To further prove that H/R exposure could trigger the activation of the proinflammatory TLR4/NF-κB signaling, we applied HUVECs as the carrier of in vitro study. Compared with the other H/R intervals, we discovered that the protein level of TLR4 was significantly higher in the H6/R12 (6 h of hypoxia and 12 h of reoxygenation) group (Fig. 6a). TLR4 expression both at the gene level (Fig. 6b) and protein level (Fig. 6c) was significantly higher in H6/R12 group than in the Control group. Additionally, H6/R12 exposure also promoted the phosphorylation of p65 (Fig. 6d). To further investigate the role of TLR4 in H/R-triggered p65 activation, we added treatment with TLR4-shRNA. We found that TLR4 interference reversed the increase of TLR4 expression (Fig. 6b, c) and the phosphorylation of p65 (Fig. 6d). These data indicated that H/R exposure significantly enhanced the activation of the proinflammatory TLR4/NF-κB signaling in HUVECs, and TLR4 interference mitigated inflammatory-related endothelial dysfunction.

## Discussion

In this study, we found that IH increased the instability of atherosclerotic plaques. After TLR4 interference, the instability of plaques was decreased with attenuated inflammation and dyslipidemia.

AS is a multifactorial systemic disease of large and medium arteries. It is gradually recognized that plaques with a rapid progression rate have a much greater probability of resulting in ACS [24]. Thin plaque fibrous caps, severe inflammatory infiltration and high lipid content are known to make plaque vulnerable [23]. OSA has been newly recognized as an underlying risk factor of AS [2, 3]. IH is the most prominent pathophysiological feature of OSA [25]. In the present study, we demonstrated that IH increased atherosclerotic plaque growth and vulnerability in ApoE^−/−^ mice, with extensive macrophage infiltration and increased lipid content, and reduced content of smooth muscle cells and collagen.

Progressive vascular and systemic chronic inflammation are the common pathways to AS plaque deterioration [26]. OSA has been proved to be related to chronic systemic inflammation and metabolic syndrome [27]. A meta-analysis has confirmed that the levels of systemic inflammatory markers, such as IL-6 and TNF-α, were higher in OSA patients compared with controls [28]. Likewise, data obtained from animal experiments also supported these results [10, 11]. In our study, IH elevated serum concentrations of IL-6 and TNF-α, and the content of IL-1β and macrophage infiltration in AS plaques in ApoE^−/−^ mice. Inflammation in atherosclerotic plaque attenuated the tensile strength of the collagen cap, enhanced cell death and reinforced prothrombotic activity, and eventually caused ACS [26]. Moreover, inflammation recruited macrophages and resulted in foam cells derived from macrophages accumulating in plaque.

NF-κB is a key transcription factor of inflammation. TLR4, a typical pattern-recognition receptor, is the upstream gene of NF-κB and has been confirmed to be closely related to inflammatory activation in plaques [15]. Accumulating pieces of evidence also showed that TLR4 is deeply implicated in activation of inflammation in patients with OSA [18].

As we’ve expected, along with increased TLR4 expression, enhanced nuclear localization of p65 and the phosphorylation of p65, and the inflammation factors were observed in the IH treated group. After TLR4 interference, vascular and systemic inflammation was greatly attenuated. Furthermore, H/R exposure promoted TLR4 expression and activation of TLR4/NF-κB signaling in endothelial cells, while TLR4 interference reversed these effects. As a result, the atherosclerotic loads and atherosclerotic plaque vulnerability were decreased more than 50% after TLR4 interference. Accordingly, we conclude that IH promotes the plaque growth and instability by triggering the activation of TLR4/NF-κB signaling and the downstream proinflammatory cascade responses. Therefore, TLR4 maybe an ideal target for interfering in the OSA-induced AS progression.

We also found IH exposure increased serum levels of total cholesterol and LDL-C. After TLR4 interference, IH-induced total cholesterol and LDL-C elevation had been significantly reversed. Previous studies reported that IH may cause atherogenesis, to a large extent, depending on dyslipidemia [10]. The IH induced dyslipidemia may play an important role in IH-mediated AS plaque progression in the present experiment. It was also reported that IH exposure down-regulates scavenger receptor class B1 (SR-B1) protein expression, disturbs cholesterol clearance and enhances cholesterol accumulation by activating NF-κB [29]. Activating NF-κB down-regulates SR-B1 and ATP-binding cassette transporter A1 (ABCA1) expression [9]. It is inferred that TLR4 interference may decrease the total cholesterol and LDL-C by inhibiting the activity of NF-κB. TLR4 signaling may also play an important role in IH-mediated AS plaque progression by aggravating dyslipidemia.

In the present study, we used ApoE^−/−^ mice as an animal model because it is more apt to show the pathological changes of OSA-accompanying AS, such as inflammatory susceptibility, dyslipidemia and extracellular matrix degradation [30–32]. To highlight the effect of IH but not a high cholesterol diet on plaque progression, we fed ApoE^−/−^ mice with a special high-fat diet (HFD), in which the content of cholesterol (0.25%) was one-fifth of the previous study (1.25%) at the beginning of our study [9, 12]. Then, mice were exposed to IH at the age of the formation of spontaneous early stage atherosclerotic lesions. This model may provide a more intuitive understanding of the process of OSA-accompanying AS development.

## Conclusions

**Fig. 7:**
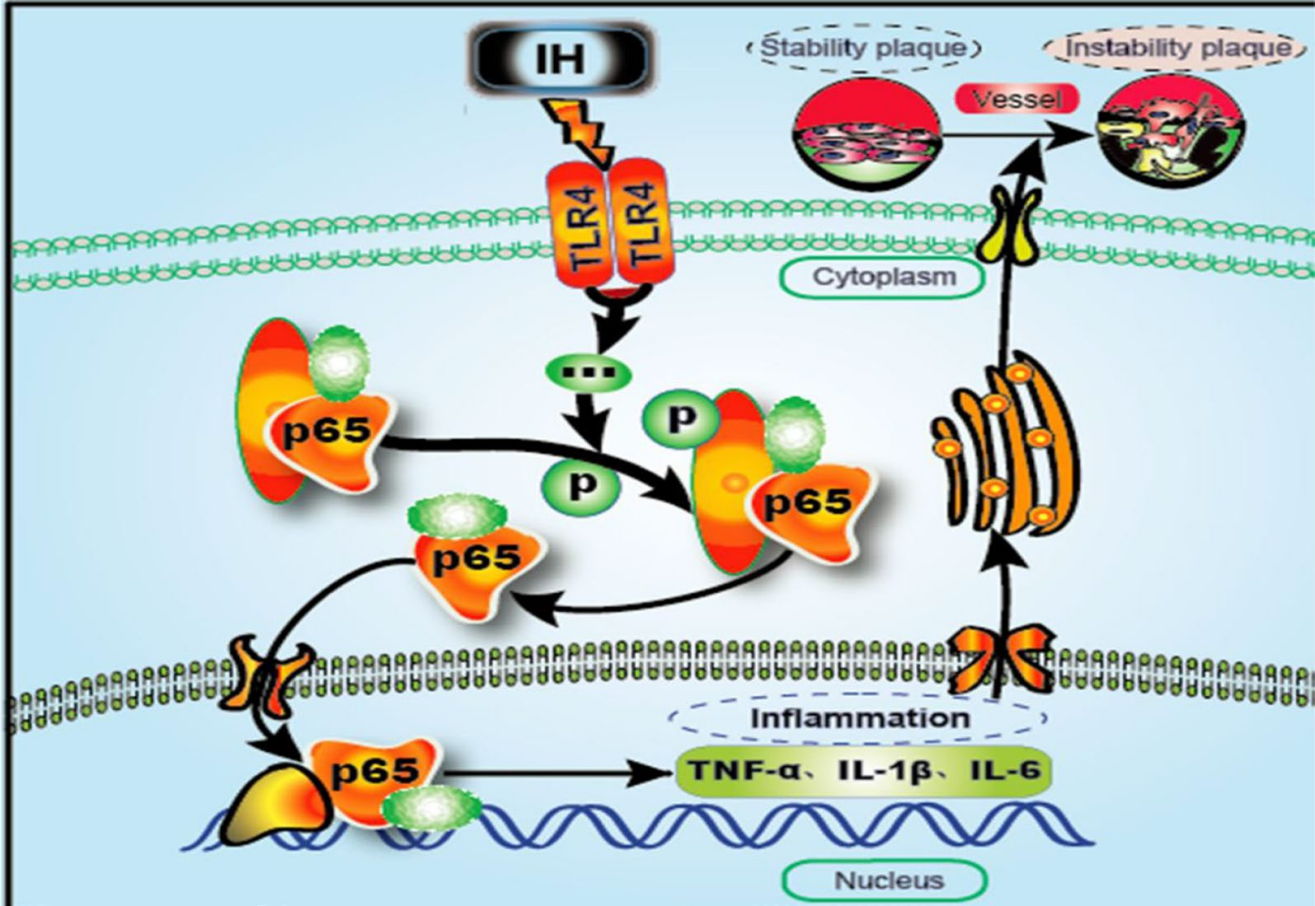
Possible mechanism diagram of IH accelerated atherosclerotic plaque growth and instability. IH exposure enhanced the expression of TLR4 and facilitated the activation of NF-κB p65 both in vivo and in vitro. Activated NF-κB entered the nucleus and augmented the expression of inflammatory cytokines. Inflammatory activity accelerated atherosclerotic plaque growth and instability. In summary, IH promotes atherosclerosis progression mainly through activating inflammatory response, which depends on TLR4/NF-κB signaling

With this modified animal model, we found that IH accelerated growth and vulnerability of atherosclerotic plaque, which probably acted by triggering the activation of TLR4/NF-κB signaling. We further prove the role of TLR4 in the H/R-triggered activation of the proinflammatory TLR4/NF-κB signaling in endothelial cells. To the best of our knowledge, it is the first report that presents substantial evidence about IH-induced AS growth and vulnerability and the detailed mechanism, in which TLR4 is involved (Fig. 7). These findings may provide a new insight into the treatment of IH-induced AS progression.

## Abbreviations

AS: atherosclerosis
OSA: obstructive sleep apnea
ACS: acute coronary syndrome
IH: intermittent hypoxia
TLR4: toll-like receptor 4
NF-κB: nuclear factor κappa B
ApoE^−/−^: apolipoprotein E-deficient
HFD: high-fat diet
EGFP: enhanced green fluorescent protein
SBP: systolic blood pressure
DBP: diastolic blood pressure
α-SMA: α-smooth muscle actin
IL-6: interleukin-6
TNF-α: tumor necrosis factor alpha
MOMA-2: macrophages/monocytes
shRNA: short hairpin RNA
H/R: hypoxia/reoxygenation
